# Mechanistic insights into the antitumor effects of astragaloside IV and astragalus polysaccharide in digestive system cancers

**DOI:** 10.3389/fphar.2025.1691011

**Published:** 2025-10-29

**Authors:** Zhenhua Cui, Qingxin Shang

**Affiliations:** College of Traditional Chinese Medicine, Shandong University of Traditional Chinese Medicine, Jinan, Shandong, China

**Keywords:** astragaloside IV, Astragalus polysaccharide, digestive system cancers, natural compounds, antitumor mechanisms

## Abstract

Malignant tumors of the digestive system are among the leading causes of cancer-related mortality worldwide. Characterized by complex pathogenesis and insidious early symptoms, these cancers remain major challenges in clinical management. With the growing interest in natural medicines, *Astragalus membranaceus* and its active components, Astragaloside IV (AS-IV) and Astragalus polysaccharide (APS), have attracted considerable attention for their therapeutic potential against digestive system malignancies. Based on more than 41 peer-reviewed studies published in recent years, this review summarizes the key antitumor mechanisms of AS-IV and APS in hepatocellular carcinoma, colorectal cancer, gastric cancer, and other digestive system malignancies. AS-IV exerts anticancer effects through the regulation of PI3K/AKT, MAPK, NF-κB, and TGF-β/Smad pathways, leading to apoptosis induction, inhibition of epithelial–mesenchymal transition, modulation of the tumor immune microenvironment, reversal of multidrug resistance, and enhancement of chemosensitivity. APS, a bioactive macromolecule with immunostimulatory and multitarget regulatory properties, enhances antitumor immunity by activating dendritic cells, promoting macrophage polarization, and suppressing immune evasion, while also improving the efficacy of chemotherapy and targeted therapies. This comprehensive review highlights molecular targets, signaling networks, and novel delivery strategies of AS-IV and APS, providing mechanistic insights and translational perspectives for their potential application in digestive system cancers.

## 1 Introduction

Malignant tumors of the digestive system refer to a group of cancers that arise in the gastrointestinal tract and associated organs, including esophageal cancer, gastric cancer, colorectal cancer, hepatocellular carcinoma, pancreatic cancer, and gallbladder cancer. These tumors have become a major global public health concern (Oduyale and Cao, 2024). They account for over 25% of all cancer incidences and approximately 35% of cancer-related deaths worldwide, reflecting both high incidence and high mortality rates (Park et al., 2025). In recent years, the incidence and mortality of digestive system malignancies have continued to rise. Their pathogenesis is highly complex, and early screening and prevention remain major clinical challenges. According to GLOBOCAN 2022 data, digestive system cancers represent a significant portion of the global cancer burden. With an estimated 1.926 million new cases, colorectal cancer ranks as the third most commonly diagnosed malignancy worldwide, followed by gastric and liver cancers, which occupy the sixth and seventh positions, respectively (Zheng Y. et al., 2025; Bray et al., 2024). In terms of cancer-related deaths, liver and gastric cancers are the third and fourth leading causes of mortality worldwide, highlighting their substantial lethality (McGlynn et al., 2025; Zhou, 2025). These cancers are more prevalent in developing countries, reflecting a pronounced disparity in cancer screening and treatment capabilities (Filho et al., 2025). In recent years, there has been a deeper understanding of malignant tumors in the medical field. Cancer is no longer viewed solely as a disease caused by abnormal cell proliferation, but rather as a complex biological system composed of tumor cells and various surrounding non-tumor cells (Yang et al., 2025). Despite the increasing diversity of therapeutic strategies-including surgery, radiotherapy, chemotherapy, targeted therapy, and immunotherapy-clinical applications still face limitations such as suboptimal efficacy, significant side effects, and high treatment costs (Yang et al., 2025; Le Cornet et al., 2025; Zhao et al., 2025). Therefore, the exploration of safer, more cost-effective, and efficacious alternative therapies has become a key direction in current cancer research.

**FIGURE 1:**
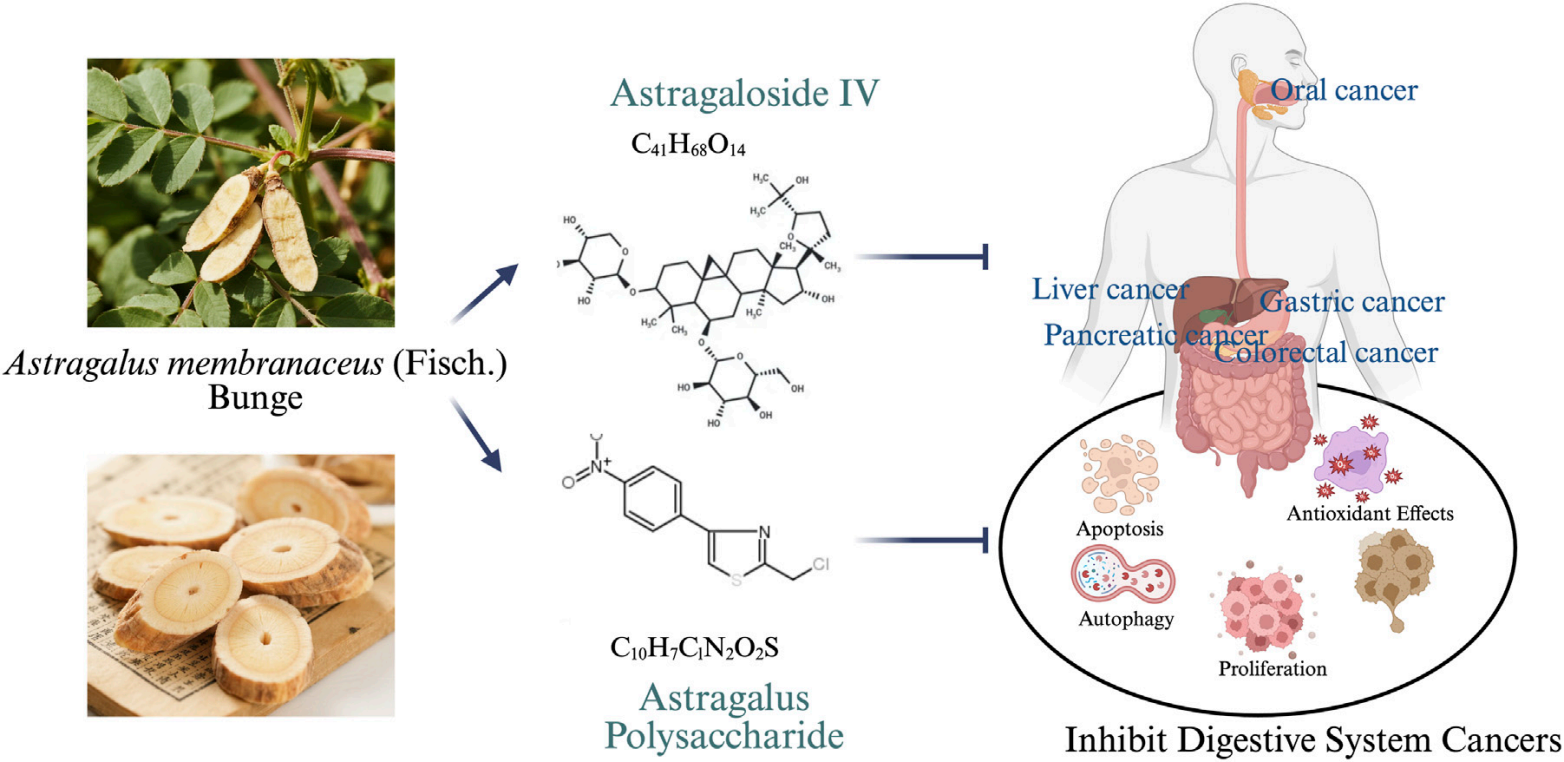
Astragaloside IV and Astragalus polysaccharide in the treatment of digestive system tumors.

*Astragalus membranaceus* (Fisch.) Bge. var. *mongholicus* (Bge.) Hsiao and *Astragalus membranaceus* (Fisch.) Bge., both belonging to the Leguminosae family, are the botanical sources of Huangqi, whose dried roots are used in traditional Chinese medicine (Wang et al., 2025). Modern pharmacological studies have demonstrated that Huangqi possesses a wide range of therapeutic properties, including immunomodulatory (Qin et al., 2025), cardiocerebrovascular protective (Yu et al., 2025), anti-inflammatory (Liu et al., 2022), antioxidant (Miao et al., 2025), antiviral (Aleebrahim-Dehkordi et al., 2022), and antidiabetic effects (Zhang et al., 2019). Moreover, Huangqi has shown remarkable efficacy in the treatment of various malignancies, such as breast cancer (Tang and Tian, 2024), lung cancer (Guo et al., 2025), gastric cancer (Wang S. et al., 2020), and ovarian cancer (Liu Y. et al., 2023). Its antitumor mechanisms are multifaceted and include the inhibition of cell proliferation, induction or promotion of apoptosis, and suppression of tumor invasion and metastasis. Huangqi contains a variety of bioactive compounds, among which flavonoids, saponins, and polysaccharides are considered the major pharmacologically active constituents (Liu et al., 2024). The saponins are primarily triterpenoid in nature and include compounds such as astragaloside, isoastragaloside, and cycloastragenol (Liu H. et al., 2025). Among them, AS-IV has been extensively studied for its potent antitumor activities, which involve inducing apoptosis, regulating the cell cycle, reversing multidrug resistance (MDR), and modulating immune responses (Wang et al., 2023). APS, the most abundant active component of Huangqi, is composed of various monosaccharides and exhibits a broad spectrum of pharmacological activities, including antitumor, anti-aging, antiviral, and lipid-lowering effects (Li C. X. et al., 2022; Li et al., 2020). This review focuses on the antitumor effects and underlying molecular mechanisms of AS-IV and APS in digestive system malignancies, aiming to provide a theoretical basis for future research and clinical application (Figure 1).

## 2 Overview of AS-IV and APS

*Astragalus membranaceus* (also known as *Astragalus mongholicus*), a member of the Fabaceae family, is one of the most commonly used qi-tonifying herbs in traditional Chinese medicine (TCM) (Hou et al., 2023). Classified as a “superior herb” in the classic text Shennong Ben Cao Jing, it has been traditionally prescribed for syndromes such as “tonifying qi and strengthening the exterior,” “promoting diuresis to reduce swelling,” and “detoxifying and promoting tissue regeneration.” Modern pharmacological studies have revealed that *Astragalus membranaceus* possesses a wide range of bioactivities, including immunomodulatory, antioxidant, anti-inflammatory, and antitumor properties, demonstrating promising clinical potential in cancer, metabolic disorders, and autoimmune diseases (Tang and Tian, 2024; Chen et al., 2022; Shan et al., 2019; Sheik et al., 2021). *Astragalus membranaceus* is rich in diverse chemical constituents, primarily flavonoids, triterpenoid saponins, polysaccharides, amino acids, and trace elements. Among these, AS-IV, APS, and various flavonoid compounds are considered the main bioactive components (Abd Elrahim Abd Elkader et al., 2022; Dong et al., 2023). AS-IV is a triterpenoid saponin with the molecular formula C_41_H_68_O_14_, typically obtained through extraction, concentration, purification, and crystallization of the plant root (Xu et al., 2024). Extensive studies have demonstrated that AS-IV exerts potent antitumor effects by inducing apoptosis and cell cycle arrest, reversing MDR, and modulating tumor immune responses, thereby highlighting its potential in cancer therapy (Chen et al., 2021; Zhou et al., 2023). Although APS contains monosaccharides such as glucose and arabinose, its antitumor activity is not attributed to direct metabolic inhibition but to immunomodulatory and signaling regulation (Jin et al., 2014). APS enhances antitumor immunity by activating macrophages, dendritic cells, and T lymphocytes, thereby promoting cytokine secretion (e.g., IFN-γ, IL-2). Furthermore, APS suppresses tumor-promoting pathways such as PI3K/AKT and NF-κB, while inducing apoptosis and reducing angiogenesis. Therefore, despite its carbohydrate composition, APS functions primarily as a biological response modifier rather than a metabolic substrate for tumor cells. Beyond its immunoregulatory effects, APS exhibits broad pharmacological activities including antiviral, anti-aging, and lipid-regulating properties, and has shown strong potential in remodeling the tumor immune microenvironment, enhancing chemosensitivity, and supporting combined immunotherapy strategies (Du et al., 2022; Shao et al., 2004). In summary, *Astragalus membranaceus* and its representative active constituents-AS-IV and APS-are emerging as promising natural candidates for modern anticancer drug development. Particularly in the context of adjuvant cancer therapy and immune microenvironment modulation, these compounds offer new perspectives for integrating traditional medicine with modern precision oncology.

## 3 Antitumor activities of AS-IV and APS in digestive system cancers

AS-IV and APS exert multifaceted antitumor effects in digestive system cancers through various mechanisms (Table 1). They effectively inhibit tumor cell proliferation, migration, invasion, and epithelial–mesenchymal transition (EMT), while promoting apoptosis and regulating the cell cycle. These compounds also reshape the tumor microenvironment by suppressing pro-tumor functions of fibroblasts, modulating immune cell polarization, and enhancing immune responses. Additionally, AS-IV and APS improve sensitivity to chemotherapeutic agents such as cisplatin, adriamycin, and apatinib by reversing drug resistance and boosting their pro-apoptotic effects. Their antitumor activity is closely associated with the regulation of oxidative stress, glycolysis, autophagy, and signaling pathways such as PI3K/Akt, NF-κB, and Wnt/β-catenin. Together, these findings highlight the potential of AS-IV and APS as promising adjunctive therapies in the treatment of digestive system malignancies.

**TABLE 1:** Summary of antitumor mechanisms of Astragaloside IV and Astragalus polysaccharide in Digestive System Cancers.

Cancer type	Bioactive compound	Representative models	Summary of core mechanisms	Key targets
Liver cancer	Astragaloside IV	Cell lines: HepG2, Huh-7; Animal: male BALB/c nude mice	Inhibits tumor cell proliferation, migration, and invasion; induces apoptosis; reverses immunosuppression and chemoresistance	TGF-β/Smad, Nrf2/HO-1, Akt/GSK-3β/β-catenin, PD-L1
	Astragalus polysaccharide	Cell lines: HepG2, H22; Animal: male BALB/c nude mice	Enhances immunomodulation; induces cell cycle arrest and apoptosis	Bax/Bcl-2, Caspase family, PD-L1
Colorectal cancer	Astragaloside IV	Cell lines: HCT116, SW480; Animal: male BALB/c nude mice	Suppresses tumor cell proliferation and epithelial-mesenchymal transition (EMT); remodels the tumor immune microenvironment; enhances chemosensitivity	IL-1β/IL-6/TNF-α, E-cadherin/N-cadherin, NOTCH3
	Astragalus polysaccharide	Cell lines HCT-116, MC38; Animal: male BALB/c nude mice	Inhibits tumor cell proliferation; activates antitumor immunity by promoting dendritic cell function	STAT3, Gal-3, CD8^+^, CD4^+^ T cells
Gastric cancer	Astragaloside IV	Cell lines: HGC-27, BGC-823; Animal: male BALB/c nude mice	Inhibits tumor cell proliferation, migration, and invasion; modulates the tumor microenvironment; reverses EMT.	E-cadherin/N-cadherin/Vimentin, miR-195-5p/PD-L1
	Astragalus polysaccharide	Cell lines: MGC-803, SGC-7901	Directly inhibits proliferation and induces apoptosis; synergizes with chemotherapeutic drugs (e.g., Doxorubicin, Apatinib) to increase sensitivity	Bax/Bcl-2, Caspase-3, p-AMPK
Pancreatic cancer	Astragalus polysaccharide	Cell lines: ASPC-1, PANC-1	Enhances the antitumor activity of apatinib against pancreatic cancer cells	VEGFR-2, MMP-9, Bax, Bcl-2, p-ERK, p-AKT, LC3
Oral cancer	Astragaloside IV	Cell lines: CAL-27, Tca8113	Enhancement of autophagy, inhibition of EMT, and suppression of tumor cell proliferation, migration, and invasion	E-cadherin, N-cadherin, α-SMA, p-AMPK, AMPK, p-AKT, AKT, p-mTOR, mTOR, LC3I, LC3II, P62

### 3.1 Antitumor mechanisms of AS-IV and APS in liver cancer

**FIGURE 2:**
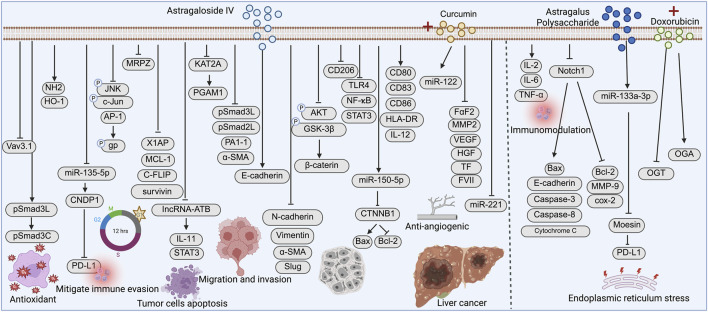
Mechanistic pathways of Astragaloside IV (AS-IV) and Astragalus polysaccharide (APS) in liver cancer. AS-IV and APS exert antitumor effects by inhibiting tumor cell migration and invasion, enhancing antioxidant defense, inducing apoptosis, improving chemosensitivity, and modulating the tumor immune microenvironment through multiple signaling pathways.

Liver cancer is the sixth most commonly diagnosed cancer and the third leading cause of cancer-related death worldwide (Cheng et al., 2022), with hepatocellular carcinoma (HCC) being the predominant histological subtype (Forner et al., 2018). Its pathogenesis is multifactorial and closely associated with chronic hepatitis B or C virus infection (HBV/HCV), alcohol abuse, non-alcoholic fatty liver disease (NAFLD), aflatoxin exposure, and liver cirrhosis (Cheng et al., 2022). Although antiviral therapies have reduced the incidence of virus-related HCC, the majority of patients are still diagnosed at intermediate or advanced stages due to the lack of early symptoms, posing a major clinical challenge (Zheng et al., 2021). In recent years, natural compounds such as AS-IV and APS have attracted increasing attention for their therapeutic potential in liver cancer (Figure 2).

#### 3.1.1 Antitumor mechanisms of AS-IV in liver cancer

AS-IV exerts its antitumor effects in HCC through multiple mechanisms. Fang Gong et al. (2023) and Zhang et al. (2021) demonstrated that AS-IV modulates the pSmad3C/3L and Nrf2/HO-1 signaling pathways, respectively, enhancing the phosphorylation of tumor-suppressive Smad3C and activating antioxidant responses to delay the progression of HCC and hepatic fibrosis. Zhu and Lu (2024) further revealed that AS-IV inhibits glycolysis and proliferation in HCC cells by blocking KAT2A-mediated succinylation of PGAM1. AS-IV also plays a critical role in immunomodulation in HCC. Ma et al. (2023) reported that AS-IV downregulates the miR-135b-5p/CNDP1 axis to suppress PD-L1 expression, thereby alleviating immunosuppression. Guo H. et al. (2022) showed that AS-IV promotes dendritic cell maturation and enhances antitumor immune responses. Similarly, Min et al. (2022) found that AS-IV inhibits the TLR4/NF-κB/STAT3 signaling pathway to prevent the polarization of tumor-associated M2 macrophages, thereby improving the immune microenvironment.

In addition, AS-IV has shown potential in reversing MDR. Wang P. P. et al. (2017) and Qu et al. (2020) demonstrated that AS-IV enhances the sensitivity of HCC cells to chemotherapeutic agents such as 5-fluorouracil (5-FU) and cisplatin by inhibiting the JNK/AP-1 signaling pathway and downregulating MRP2 expression, respectively. Notably, AS-IV also mitigated cisplatin-induced nephrotoxicity. In terms of apoptosis induction, Su et al. (2020) found that AS-IV activates both intrinsic and extrinsic apoptotic pathways, induces G1 cell cycle arrest, and suppresses HCC cell growth. Li et al. (2018) reported that AS-IV downregulates lncRNA-ATB, inhibits the IL-11/STAT3 axis, and thereby suppresses EMT and tumor metastasis. Moreover, Qin et al. (2017) demonstrated that AS-IV inhibits the Akt/GSK-3β/β-catenin signaling pathway, reducing migration and invasion of HCC cells.

At the molecular level, Cui et al. (2020) revealed that AS-IV upregulates miR-150-5p to suppress CTNNB1 expression and induce apoptosis in HCC cells. Qi et al. (2010), using proteomic analysis, found that AS-IV downregulates the oncogene Vav3.1, contributing to its antitumor activity. Furthermore, Zhang et al. (2017) investigated the synergistic effects of AS-IV combined with curcumin in HCC, showing enhanced antitumor and antiangiogenic efficacy through downregulation of VEGF, MMP2, and FGF2, and modulation of miR-122 and miR-221 expression.

#### 3.1.2 Antitumor mechanisms of APS in liver cancer

APS has also demonstrated multiple antitumor mechanisms in liver cancer. In H22 tumor-bearing mice, Lai et al. (2017) observed that APS enhances immune organ indices, increases IL-2, IL-6, and TNF-α levels, and promotes apoptosis by regulating Bcl-2/Bax expression. He et al. (2022) further showed that APS upregulates miR-133a-3p to inhibit Moesin expression, thereby downregulating PD-L1 and alleviating immune evasion. Huang et al. (2016) reported that APS suppresses the Notch1 signaling pathway and inhibits tumor growth and metastasis by modulating apoptotic factors such as caspase-3 and caspase-8. Li et al. (2023) proposed that APS enhances endoplasmic reticulum stress and apoptosis when combined with doxorubicin, via modulation of the O-GlcNAc protein modification pathway.

In the field of nanomedicine, Ji et al. (2023) and Jiao et al. (2022) synthesized APS-based selenium nanoparticles (AASP-SeNPs and APS-SeNPs), both of which exhibited good water solubility, stability, and significant antiproliferative effects on HepG2 cells. These nanoparticles induced apoptosis via mitochondrial pathways, suggesting their potential as targeted therapeutic agents for liver cancer.

### 3.2 Antitumor mechanisms of AS-IV and APS in colorectal cancer

**FIGURE 3:**
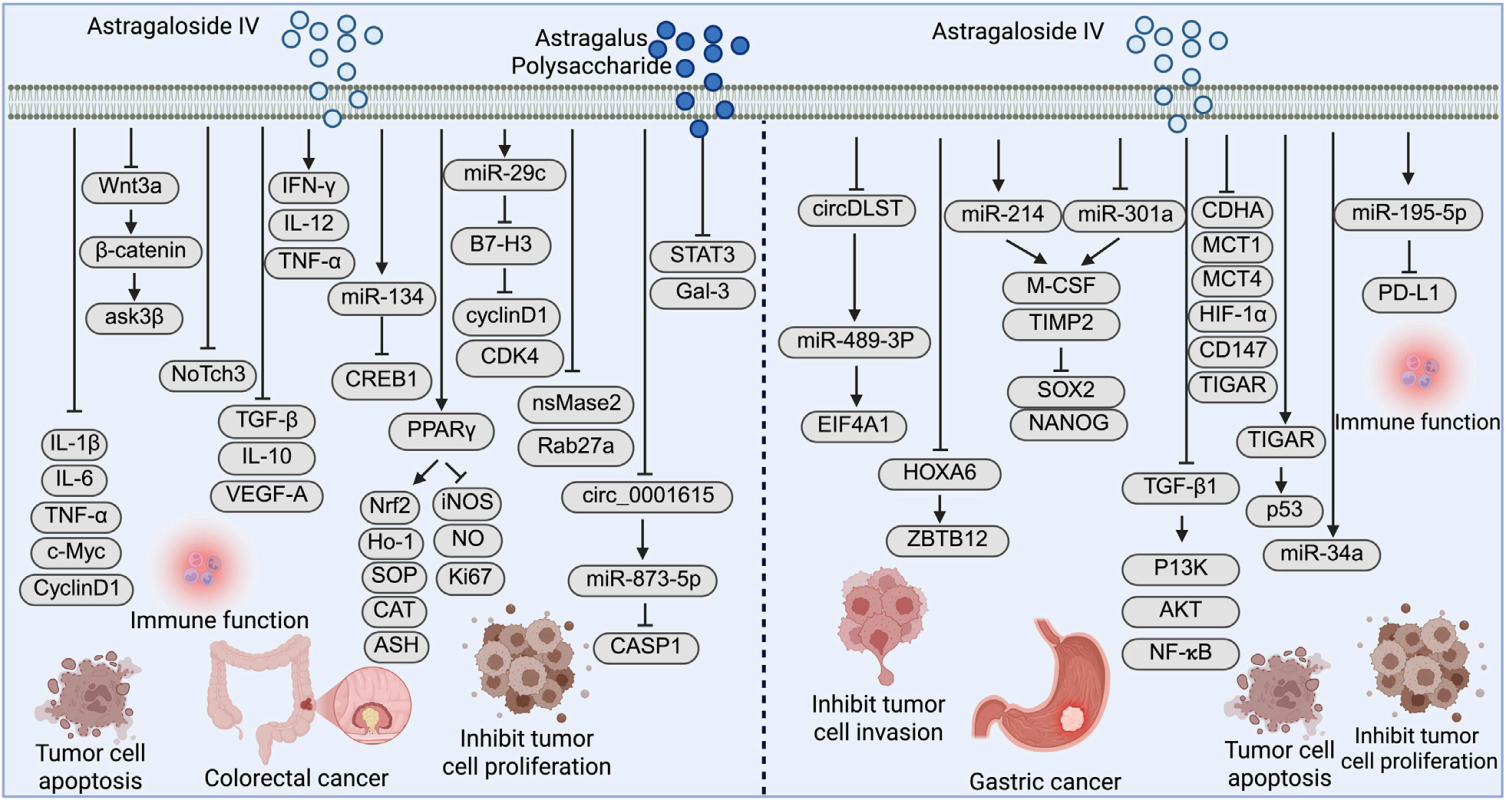
Mechanistic pathways of Astragaloside IV (AS-IV) and Astragalus polysaccharide in colorectal cancer, and AS-IV in gastric cancer: promoting tumor cell apoptosis, inhibiting proliferation, migration, and invasion, and modulating the tumor immune microenvironment.

Colorectal cancer is the third most commonly diagnosed cancer and the second leading cause of cancer-related death worldwide (Dekker et al., 2019). In recent years, its incidence has been steadily increasing, with a particularly notable rise among younger populations (Siegel et al., 2020). The development of colorectal cancer is closely linked to various factors, including genetic susceptibility, lifestyle habits, chronic intestinal inflammation, and gut microbiota dysbiosis (Abedizadeh et al., 2024; Li et al., 2024a). Despite advances in screening techniques and therapeutic approaches-such as colonoscopy, targeted therapy, and immune checkpoint inhibitors-the prognosis of colorectal cancer remains suboptimal, largely due to the lack of early specific symptoms, which leads to diagnosis at advanced or metastatic stages in many patients (Bresalier, 2022; Jackson et al., 2024; Schlechter and Ng, 2022). Against this background, natural products such as AS-IV and APS, with their multitarget activity, low toxicity, immunomodulatory and antitumor properties, have gained increasing attention in colorectal cancer prevention and therapy (Figure 3).

#### 3.2.1 Antitumor mechanisms of AS-IV in colorectal cancer

AS-IV has demonstrated significant antitumor activity in multiple *in vitro* and *in vivo* models of colorectal cancer. Wen et al. (2024) reported that AS-IV markedly reduced colonic adenoma formation in high-fat diet-induced Apc^Min/+ mice. The underlying mechanisms included modulation of the gut microbiota, inhibition of the Wnt/β-catenin signaling pathway, and downregulation of pro-inflammatory cytokines and tumor markers. Interestingly, germ-free conditions weakened the antitumor effects of AS-IV, while probiotic transplantation restored its efficacy, suggesting a pivotal role of the intestinal microbiota in its mechanism of action.

Xie et al. (2016) were the first to show that AS-IV enhances cisplatin sensitivity in colorectal cancer by suppressing the NOTCH3 signaling pathway, thereby overcoming partial chemoresistance. In terms of immune modulation, Liu et al. (2020) demonstrated that AS-IV promotes M1 macrophage polarization and suppresses M2 macrophages. When combined with the immune checkpoint inhibitor anti-PD-1, it synergistically enhanced T cell infiltration and antitumor activity. Moreover, AS-IV was found to upregulate miR-134, suppress EMT, increase sensitivity to oxaliplatin, and downregulate CREB1 expression, thereby reducing colorectal cancer cell migration, invasion, and chemoresistance (Ye et al., 2017). In the azoxymethane/dextran sulfate sodium (AOM/DSS)-induced colitis-associated cancer (CAC) model, AS-IV activated the PPARγ/Nrf2 signaling pathway, restored antioxidant enzyme levels, inhibited reactive oxygen species (ROS)/NO accumulation, and reduced DNA damage, ultimately preventing tumor initiation (Liang et al., 2023). Wang et al. (2018) further confirmed that AS-IV upregulates miR-29c to inhibit the immune checkpoint molecule B7-H3, resulting in G0/G1 cell cycle arrest and suppression of colorectal cancer cell proliferation. Zhou et al. (2024) revealed that AS-IV reduces tumor-derived extracellular vesicle (TEV) release by downregulating nSMase2 and Rab27a, thereby preventing TEV-induced M2 macrophage polarization and inhibiting liver metastasis of colorectal cancer. In addition, Kong et al. (2024) found that AS-IV downregulates circ_0001615 and LASP1 while activating miR-873-5p expression, thus exerting antitumor effects via the circ_0001615/miR-873-5p/LASP1 regulatory axis.

#### 3.2.2 Antitumor mechanisms of APS in colorectal cancer

APS has shown potent efficacy in alleviating inflammation-induced immunosuppression. Li et al. (2024b) demonstrated that APS activates CD8^+^ T cells and reverses their functional exhaustion by targeting the STAT3/Galectin-3/LAG3 pathway, thereby significantly inhibiting tumor growth in inflammation-driven colorectal cancer mouse models. Tamang et al. (2022) developed a novel polysaccharide by cultivating Lentinula edodes (shiitake mushroom) on Astragalus substrate. The extracted polysaccharide, particularly under ultrasound-enzyme-assisted extraction (UEA) conditions, exhibited enhanced antiproliferative activity against HCT116 colorectal cancer cells, indicating the potential of integrating APS with functional dietary interventions. Furthermore, Cao et al. (2024) designed a nanoparticle vaccine (NP-TCL@APS) co-loaded with APS and colorectal cancer tumor cell lysates. This vaccine significantly activated dendritic cells, enhanced the antigen-specific cytotoxicity of CD8^+^ T cells, and effectively inhibited tumor growth, showing good biocompatibility and promising potential for cancer immunotherapy.

### 3.3 Molecular mechanisms of AS-IV and APS in gastric cancer

Gastric cancer ranks as the fifth most common malignancy and the fourth leading cause of cancer-related deaths worldwide, with particularly high incidence rates in East Asia (Smyth et al., 2020; López et al., 2023). Its pathogenesis is multifactorial, closely associated with *Helicobacter pylori* infection, chronic gastritis, dietary habits, smoking, genetic predisposition, and gastric mucosal atrophy (Duan et al., 2025; Karimi et al., 2014). Although advances in endoscopic screening and targeted therapies have improved early detection and outcomes in some patients, the majority are still diagnosed at advanced stages, resulting in poor 5-year survival rates and presenting significant clinical challenges (Oliveira et al., 2015; Shah and Bentrem, 2024; Sun et al., 2025). In recent years, AS-IV and APS have drawn increasing attention due to their favorable safety profiles and antitumor activities, showing great potential in the prevention and treatment of gastric cancer.

#### 3.3.1 Antitumor mechanisms of AS-IV in gastric cancer

Recent studies have increasingly revealed the multitarget antitumor potential of AS-IV in gastric cancer (Figure 3). Li F. et al. (2022) demonstrated that AS-IV suppresses the expression of circDLST in gastric cancer cells, thereby disrupting its “sponge” effect on miR-489-3p. This leads to reduced expression of EIF4A1, ultimately inhibiting cancer cell proliferation and metastasis. These effects were further validated in xenograft models, suggesting that the circDLST/miR-489-3p/EIF4A1 axis may represent a critical therapeutic target of AS-IV. Liu H. et al. (2023) further elucidated the role of AS-IV in modulating the tumor microenvironment of gastric cancer. Their study showed that high expression of HOXA6 and ZBTB12 in cancer-associated fibroblasts (CAFs) promotes tumor progression, while AS-IV inhibits the transcriptional activity of HOXA6, downregulates ZBTB12, and suppresses gastric cancer cell proliferation and migration. These findings suggest that AS-IV may regulate tumor–stroma interactions through targeting CAFs. In addition, Wang Z. F. et al. (2017) reported that AS-IV regulates the expression of miR-214 and miR-301a in gastric CAFs, reduces the secretion of the pro-tumorigenic cytokine M-CSF, and increases the expression of the anti-tumor factor TIMP2. This leads to the downregulation of stemness markers such as SOX2 and NANOG in cancer cells, thereby exerting antitumor effects.

AS-IV has also been shown to reverse EMT induced by TGF-β1. Zhu and Wen (2018) found that AS-IV inhibits the PI3K/Akt/NF-κB signaling pathway, thereby blocking EMT and reducing gastric cancer cell invasion and metastasis, indicating its therapeutic potential in metastasis prevention. In the context of precancerous gastric lesions, Zhang et al. (2018) used a rat model of precancerous lesions of gastric cancer (PLGC) induced by MNNG and found that AS-IV downregulated glycolysis-related enzymes (LDHA, MCT1, HIF-1α) while upregulating miR-34a and p53 expression. These findings suggest that AS-IV may prevent gastric cancer development by regulating metabolic homeostasis and reversing mucosal dysplasia. Regarding immune-related mechanisms, Liu et al. (2021) reported that AS-IV upregulates miR-195-5p, which directly targets and inhibits the immune checkpoint molecule PD-L1. This leads to the blockade of EMT and angiogenesis and enhances immune-mediated tumor clearance, supporting the role of AS-IV as a potential immunomodulator in gastric cancer therapy.

#### 3.3.2 Antitumor mechanisms of APS in gastric cancer

**FIGURE 4:**
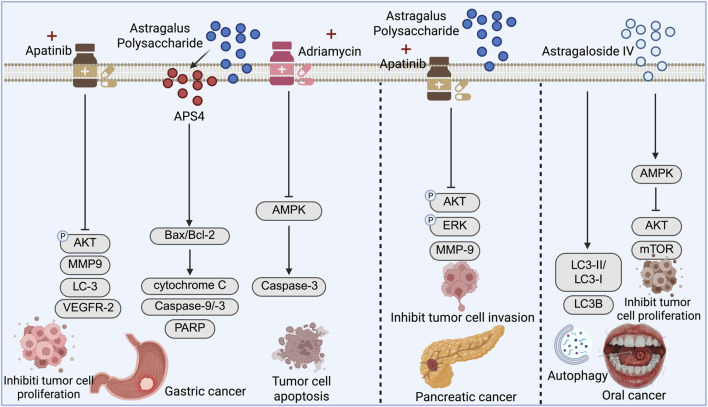
Mechanistic pathways of Astragalus polysaccharide (APS) in gastric and pancreatic cancers, and Astragaloside IV (AS-IV) in oral cancer: inhibiting tumor cell proliferation, migration, and invasion, while promoting apoptosis and autophagy.

APS has also demonstrated broad-spectrum antitumor potential in gastric cancer (Figure 4). APS can be classified into multiple subtypes depending on the isolation method. For example, based on molecular weight, APS can be divided into components such as APS1 (<10 kDa) and APS2 (10–50 kDa), with APS1 exhibiting stronger prebiotic activity and enhanced intestinal barrier repair in the context of ulcerative colitis. Meanwhile, components obtained via graded ethanol precipitation, such as APS3 (15.3 kDa), possess a complex monosaccharide composition and demonstrate significant immunomodulatory activity (Jiang et al., 2016; Huo et al., 2024). Yu et al. (2019) further isolated a novel cold water-soluble polysaccharide, APS4, from *Astragalus membranaceus*, which markedly inhibited the proliferation of gastric cancer MGC-803 cells. APS4 induced S-phase cell cycle arrest and mitochondrial pathway-mediated apoptosis, characterized by ROS accumulation, decreased mitochondrial membrane potential, an elevated Bax/Bcl-2 ratio, and activation of the caspase cascade.

In terms of chemosensitization, Wu et al. (2018a) showed that APS enhanced the antiproliferative and pro-apoptotic effects of apatinib on gastric cancer AGS cells. The underlying mechanism was associated with inhibition of the AKT signaling pathway and modulation of autophagy. The combination therapy significantly potentiated antitumor efficacy, providing a novel strategy for utilizing APS as an adjuvant to chemotherapy. Additionally, APS was shown to induce apoptosis in gastric cancer cells via activation of the AMPK pathway and to increase the sensitivity of cells to doxorubicin (Song et al., 2020). Mechanistically, APS treatment significantly upregulated the expression of tumor suppressor genes such as SEMA3F, P21, and FBXW7, while having minimal impact on MDR genes such as MDR1. These findings suggest that APS may serve as an effective chemosensitizer, enhancing therapeutic outcomes and offering promising potential for clinical application in gastric cancer management (Figure 4).

### 3.4 Emerging antitumor dechanisms of AS-IV and APS in other digestive system cancers

Pancreatic cancer ranks as the twelfth most common cancer globally but is the fifth leading cause of cancer-related death (Cai et al., 2021; Klein, 2021). It is characterized by high aggressiveness and extremely poor prognosis, with pancreatic ductal adenocarcinoma (PDAC) being the most prevalent subtype (Dhillon and Betancourt, 2020). Due to the pancreas’s deep anatomical location and the absence of specific early symptoms, most patients are diagnosed at advanced stages or with distant metastases, resulting in a 5-year survival rate of less than 10% (Wang et al., 2019).

Wu et al. (2018b) demonstrated that both ASP-C1 and PANC-1 pancreatic cancer cell lines express vascular endothelial growth factor receptor 2 (VEGFR-2). Treatment with apatinib and APS significantly inhibited cell proliferation in a dose-dependent manner. Combination therapy showed a more pronounced effect in suppressing cell migration and invasion and significantly increased apoptotic rates. Mechanistic studies revealed that the combined treatment was more effective than monotherapy in downregulating the phosphorylation of AKT and ERK and reducing the expression of matrix metalloproteinase-9. Both agents also induced autophagy, although combination treatment did not further enhance the expression of autophagy-related proteins. This study is the first to reveal that APS can enhance the antitumor effects of apatinib in pancreatic cancer, providing experimental evidence for its use as an adjuvant therapeutic agent.

Oral cancer is a malignancy with a complex pathogenesis, primarily associated with chronic tobacco use, excessive alcohol consumption, poor oral hygiene, human papillomavirus (HPV) infection, and persistent mucosal irritation (Valdez and Brennan, 2018; Paré and Joly, 2017). Due to the concealed nature of early lesions and nonspecific clinical manifestations, most patients are diagnosed at intermediate or advanced stages, often with lymph node metastasis and local recurrence, significantly impacting survival rates and quality of life (Hunter and Yeoman, 2013).

Yin et al. (2024) found that AS-IV exerts a significant dose-dependent inhibitory effect on oral cancer cells, effectively suppressing their proliferation, migration, and invasion. Mechanistically, AS-IV enhances autophagic activity-evidenced by an increased LC3II/LC3I ratio and elevated LC3B fluorescence-and inhibits the EMT, thereby exerting antitumor effects. Further investigation showed that AS-IV activates the AMPK signaling pathway while inhibiting the AKT/mTOR pathway, thereby coordinately regulating autophagy and EMT to suppress malignant progression in oral cancer cells. These findings provide strong theoretical support for the potential application of AS-IV in the treatment of oral cancer (Figure 4).

## 4 Advances in derivatives and novel formulations of AS-IV and APS for digestive system cancers

In recent years, increasing efforts have been made to enhance the therapeutic efficacy and translational potential of AS-IV and APS in the treatment of digestive system malignancies through structural modification and formulation optimization. Due to the low water solubility and bioavailability of AS-IV, structural modifications have focused on glycoside or hydroxyl group sites, such as the introduction of hydrophilic polymers or lipophilic side chains, to improve its *in vivo* stability and tumor-targeting ability (Chen et al., 2023). While studies on APS derivatives targeting gastrointestinal tumors remain limited, existing modification strategies in breast and lung cancers offer valuable references for future research (Liu M. et al., 2025; Guo C. et al., 2022; Wang B. et al., 2020; Xiong et al., 2020).

As a structurally complex and heterogeneous polysaccharide, APS contains components with varying bioactivities. Researchers have isolated and purified fractions with specific molecular weights or branched-chain structures, such as APS-I, APS-II, and APS4, to identify the most biologically active constituents (Chen et al., 2023; Zheng H. et al., 2025). For example, APS4, a cold water-soluble polysaccharide isolated by Yu et al. (2019), significantly inhibited the proliferation of gastric cancer MGC-803 cells and induced mitochondrial-mediated apoptosis.

To overcome issues such as poor *in vivo* stability and low bioavailability of AS-IV and APS, a range of novel delivery systems have been developed. Cao et al. (2024) constructed a PLGA-based nanovaccine (NP-TCL@APS), co-loaded with APS and tumor cell lysates, which not only facilitated antigen delivery but also effectively activated CD8^+^ T-cell-mediated immune responses, showing promising therapeutic potential against colorectal cancer. In addition to nanoparticles, AS-IV has also been incorporated into liposomes, microspheres, and nanogels to improve its targeted delivery and sustained efficacy in liver and gastric cancers.

APS, as a bioactive constituent of traditional Chinese medicine, is also widely used in modern combinational cancer therapies. For instance, APS significantly enhanced the inhibitory effects of apatinib on VEGFR-2–positive cells in gastric and pancreatic cancers, potentially through inhibition of the AKT/ERK signaling pathways and induction of autophagy (Wu et al., 2018a; Wu et al., 2018b). Moreover, Tamang et al. (2022) developed a co-culture strategy by growing *Lentinula edodes* (shiitake mushrooms) on an *Astragalus* substrate. The extract from this “Astragalus-mushroom” combination showed stronger antiproliferative activity in HCT116 colorectal cancer cells, demonstrating the potential of food-medicine homologous co-culture approaches in cancer therapy.

AS-IV and APS have demonstrated promising antitumor effects in various digestive system malignancies, including hepatocellular carcinoma, gastric cancer, colorectal cancer, and pancreatic cancer. Their mechanisms involve inhibition of tumor cell proliferation, induction of apoptosis, reversal of EMT, modulation of the tumor immune microenvironment, and enhancement of radiochemotherapy sensitivity. However, several challenges remain, including unclear pharmacophores of certain derivatives, a lack of systematic pharmacokinetic and toxicological evaluations, and technical hurdles in the large-scale preparation and biodegradability of some nanoparticle formulations. Future research should focus on interdisciplinary collaboration to accelerate the development of efficient delivery systems, structural optimization, and comprehensive preclinical evaluation frameworks. These efforts will lay a solid foundation for the translational application of AS-IV and APS in the treatment of digestive system malignancies.

## 5 Discussion

Digestive system malignancies represent a major global health burden due to their high incidence and mortality rates (Hao et al., 2024). Despite the availability of diverse treatment strategies, including surgery, chemotherapy, radiotherapy, targeted therapy, and immunotherapy, these approaches are often associated with limited efficacy, severe side effects, and high economic costs (Paleari, 2024; Passaro et al., 2024). Consequently, safer, more effective, and affordable alternative therapies are urgently needed. Among natural products derived from traditional Chinese medicine, Astragalus membranaceus–derived compounds, particularly AS-IV and APS, have emerged as promising candidates for digestive system cancer therapy.

Evidence from *in vitro* studies consistently demonstrates that AS-IV and APS inhibit tumor cell proliferation, migration, invasion, and EMT, while inducing apoptosis and cell cycle arrest. AS-IV regulates key signaling pathways such as Smad3, Nrf2, NF-κB, and STAT3, modulates non-coding RNA axes including miR-135b-5p/CNDP1 and circDLST/miR-489-3p/EIF4A1, and influences glycolysis and metabolic homeostasis. APS exerts antitumor effects through STAT3, Notch1, AMPK, and various miRNA networks, activating mitochondrial apoptosis and antigen-specific immune responses. Both compounds exhibit synergistic effects with chemotherapeutic agents such as 5-fluorouracil, cisplatin, apatinib, and doxorubicin, as well as with natural compounds like curcumin.

*In vivo* studies confirm that AS-IV and APS suppress tumor growth, modulate the tumor immune microenvironment, and enhance chemosensitivity in animal models of liver, colorectal, gastric, pancreatic, and oral cancers. Mechanistically, AS-IV promotes M1 macrophage polarization, suppresses PD-L1 expression, and modulates cancer-associated fibroblast function, whereas APS enhances CD8^+^ T cell activation and reverses immunosuppression. Gut microbiota–dependent models indicate that intestinal microbial modulation contributes to AS-IV’s efficacy in colorectal cancer prevention.

In silico studies and novel formulations have further explored the translational potential of these compounds. Molecular docking and network pharmacology studies have identified multiple targets and signaling pathways, while nanoparticle-based delivery systems, PLGA microspheres, selenium nanoparticles, and nanovaccines improve bioavailability, tumor targeting, and therapeutic efficacy.

An integrative analysis across cancer types reveals that immune modulation, apoptosis induction, and chemosensitization are the most consistently observed mechanisms of AS-IV and APS, whereas metabolic regulation and specific signaling pathway modulation show cancer-type specificity. In liver cancer, AS-IV consistently induces apoptosis, inhibits glycolysis, and reverses MDR, while APS modulates immune responses and enhances chemotherapy efficacy. In colorectal cancer, AS-IV strongly inhibits Wnt/β-catenin signaling, reverses EMT, and modulates gut microbiota, whereas APS activates CD8^+^ T cell–mediated immunity and serves as an adjuvant in nanoparticle-based vaccines. In gastric cancer, both AS-IV and APS regulate circRNA/miRNA axes, CAF function, and EMT, and enhance chemotherapy sensitivity. Evidence for pancreatic and oral cancers is limited but indicates promising effects through autophagy regulation, apoptosis induction, and AKT/AMPK pathway modulation.

Despite encouraging preclinical findings, several challenges remain. Most studies are limited to *in vitro* or animal models, with insufficient pharmacokinetic, toxicological, and safety evaluations to support clinical translation. AS-IV suffers from poor bioavailability, APS exhibits structural heterogeneity, and large-scale production and stability of nanoparticle formulations remain technical hurdles. Additionally, the complexity of intersecting molecular targets and signaling networks necessitates further mechanistic studies using multi-omics approaches to identify key regulatory nodes and biomarkers.

In conclusion, AS-IV and APS exhibit remarkable antitumor activities across digestive system malignancies through multifaceted mechanisms, including apoptosis induction, modulation of metabolic pathways, reprogramming of the tumor immune microenvironment, and enhancement of chemosensitivity. To advance clinical translation, future research should focus on elucidating precise molecular targets and signaling pathways, developing efficient and safe delivery systems and combination strategies, and conducting systematic preclinical and early-phase clinical studies. AS-IV and APS represent promising anticancer candidates that bridge traditional medicinal knowledge with modern precision therapeutics, offering innovative avenues for integrated prevention and treatment of digestive system cancers.
