# Supplementary material for: Mechanistic insights into the antitumor effects of astragaloside IV and astragalus polysaccharide in digestive system cancers
Source: Front Pharmacol. 2025 Oct 29;16:1691011. doi: 10.3389/fphar.2025.1691011 (PMC12605028; doi:10.3389/fphar.2025.1691011)
Supplement: Supplementary file 1 [file Table1.docx]

Supplementary Table 1 Mechanism of action of Astragaloside IV in Digestive System Cancers.

| Cancer | Real modules (animal/cell) | Possible mechanisms | Targets | Doses | Ref. |
| --- | --- | --- | --- | --- | --- |
| Liver cancer | Animal: pSmad3C^+/−^ and Nrf2^− /−^ mice  Cell: plasmid - or lentivirus - transfected HepG2 cells | Antioxidant, Anti-proliferative, Anti-migratory/invasive | Nrf2, HO-1, pSmad3C/3L, TGF-β1, p21 | 40 mg/kg, 20 μmol/L | [40] |
|  | Animal: DEN/CCl_4_/C_2_H_5_OH mice  Cell: HSC-T6/HepG2 cell | Anti-fibrotic and anti-hepatocarcinogenic effects | TGF-β1, pSmad3C, pSmad3L, pNrf2, pSmad2C, pSmad2L, PAI-1, Nrf-2, HO-1, NQO1 | 40 mg/kg, 20 μmol/L | [41] |
|  | Cell: SNU-182, Huh7 cell | Inhibition of glycolysis and tumor cell metabolic proliferation | KAT2A, KAT3B, PGAM1, CPT1A, SIRT5, SIRT7 | 20 µg/mL | [42] |
|  | Cell: Huh-7, SMMC-7721 cell | Suppresses tumor growth and mitigates immune evasion | miR-135b-5p, CNDP1, PD-L1 | 80 µg/mL | [43] |
|  | Cell: SMMC-7721 cell | Enhances dendritic cell function and anti-liver cancer immunity | CD40, CD80, CD83, CD86, HLA-DRIL-12 | 100 µg/mL | [44] |
|  | Cell: THP-1, Huh-7 cells  Animal: male BALB/c-nude mice | Suppresses M2 macrophage polarization, modulates immune microenvironment, and reverses MDR | TLR4, NF-κB, STAT3 | 120 μmol/l, 40, 80, and 100 mg/kg | [45] |
|  | Cell: Bel-7402/FU cell | Reverses MDR in HCC cells | p-JNK, c-Jun, AP1 | 11 μmol/L | [46] |
|  | Cell: HepG2, H22 cell  Animal: Balb/c mice | Enhances cisplatin sensitivity in HCC and reduces side effects | MRP2 | 0.4, 4 and 40 μmol/L, 50 mg/kg | [47] |
|  | Cell: SK-Hep, Hep3B cell | Induces apoptosis and programmed death of HCC cells; inhibits proliferation and invasion | XIAP, MCL1, C-FLIP | 200，400 μmol/L | [48] |
|  | Cell: SMMC-7721, huh-7 cell | Inhibits tumor EMT and migratory ability, while promoting tumor cell apoptosis | lncRNA-ATB, IL-11, STAT3 | 80 μg/ml | [49] |
|  | Cell: Huh7, MHCC97-H cell | Inhibits tumor cell migration and invasion | Akt, GSK-3β, β-catenin, E-cadherin, N-cadherin, vimentin, α-SMA, Slug | 10, 50, 100 μg/ml | [50] |
|  | Cell: SMMC-7721, huh-7 cell  Animal: male BALB/c nude mice | Induces apoptosis in tumor cells | miR-150-5p, CTNNB1, Bax, Bcl-2, β-catenin | 20 μg/ml | [51] |
|  | Cell: HepG2 cell | Downregulates key oncogenic protein expression, thereby inhibiting hepatocellular carcinoma | Vav3.1 | 150 μg/ml | [52] |
|  | Cell: HepG2 cell  Animal: male BALB/c nude mice | Synergistic antitumor and anti-angiogenic effects in hepatocellular carcinoma when combined with curcumin | FGF2, MMP2, VEGF, HGF, TF, FVII, miR-221 | 20 mg/kg | [53] |
| Colorectal cancer | Cell: HT29, HCT116, SW620  Animal: Male C57BL/6J *Apc^Min/+^* mice | Inhibition of local inflammatory factors in the colon and suppression of tumor cell proliferation | IL-1β, IL-6, TNF-α, c-Myc, Cyclin D1 | 50 mg/kg | [67] |
|  | Cell: HCT116, SW480 cell | Enhanced tumor cell sensitivity to cisplatin | NOTCH3 | 7.5 ng/ml | [68] |
|  | Cell: CT26 cell  Animal: BALB/c female mouse | Improved tumor immune microenvironment, inhibited tumor cell proliferation, and induced apoptosis | Arg1, Mrc1, IL12, Nos2 | 100nm, 15.0 mg/kg | [69] |
|  | Cell: SW480 cell | Inhibited the EMT process, enhanced oxaliplatin sensitivity, and suppressed tumor cell migration and invasion | miR-134, CREB1, E-cadherin, N-cadherin, Snail, Vimentin | 10 μg/ml | [70] |
|  | Animal: C57BL/6J male mice  Cell: IEC-6 | Restored antioxidant enzyme levels and reduced DNA damage | PPARγ, Nrf2, iNOS, HO-1 | 80 mg/kg | [71] |
|  | Cell: SW620, HCT116 cell | Regulated cell cycle to inhibit tumor cell proliferation | cyclin D1, CDK4, B7-H3, miR-29c | 50,100 ng/ml | [72] |
|  | Cell: MC38 cell Animal: C57BL/6J mice | Tumor microenvironment remodeling and regulation of immune cell polarization | nSMase2, Rab27a | 12.5, 25 and 50 μM, 50 mg/kg | [73] |
|  | Cell: LoVo, HCT-116  Animal: BALB/C nude mice | Inhibition of tumor cell proliferation, promotion of apoptosis, and suppression of migration | Bax, MMP9, LASP1, miR-873-5p | 200ng/mL, 15mg/kg | [74] |
| Gastric cancer | Cell: HGC-27, MKN-45 cell  Animal: Nude mice | Inhibition of gastric cancer cell proliferation and metastasis | E-cad, N-cad, Vimentin, EIF4A1, miR-489-3p | 10, 20, 40 μg/ml | [85] |
|  | Cell: MGC803, BGC-823 cell | Modulation of the tumor microenvironment to inhibit tumor cell growth, migration, and invasion | α-SMA, Vimentin, FSP, S100A4, HOXA6, ZBTB12 | 40 μm/L | [86] |
|  | Cell: BGC-823 cell | Inhibition of the pro-tumorigenic functions of fibroblasts within the tumor microenvironment | miR-214, miR-301a, SOX2, NANOG | 40 μm/L | [87] |
|  | Cell: BGC‐823, MKN‐74 | Inhibition of gastric cancer cell viability, invasion, and migration capabilities | E‐cadherin, N‐cadherin, Vimentin, MMP-2, MMP-9, Snail, p-AKT, p-p65 | 10, 20μg/ml | [88] |
|  | Animal: Male Sprague–Dawley rats | Regulation of glycolysis to alleviate abnormal metabolic conditions in precancerous gastric lesions | LDHA, p53, TIGAR, CD147, MCT1, MCT4, HIF‐1α | 50,100 mg/kg | [89] |
|  | Cell: SGC7901, MGC803 | Inhibition of EMT and angiogenesis in gastric cancer | miR-195-5p, PD-L1, E-cadherin, N-cadherin, Snail, vimentin | 10 μg/mL | [90] |
| Oral cancer | Cell: CAL-27, Tca8113 cell | Enhancement of autophagy, inhibition of EMT, and suppression of tumor cell proliferation, migration, and invasion | E-cadherin, N-cadherin, α-SMA, p-AMPK, AMPK, p-AKT, AKT, p-mTOR, mTOR, LC3I, LC3II, P62 | 25, 50 and 100 μM | [104] |
