# Supplementary material for: Mechanistic insights into the antitumor effects of astragaloside IV and astragalus polysaccharide in digestive system cancers
Source: Front Pharmacol. 2025 Oct 29;16:1691011. doi: 10.3389/fphar.2025.1691011 (PMC12605028; doi:10.3389/fphar.2025.1691011)
Supplement: Supplementary file 2 [file Table2.docx]

Supplementary Table 2 Mechanism of action of Astragalus Polysaccharide in Digestive System Cancers.

| Cancer | Modules (animal/cell) | Possible mechanisms | Targets | Doses | Ref |
| --- | --- | --- | --- | --- | --- |
| Liver cancer | Animal: Kunming mice  Cell: H22 cell | Enhanced immunomodulation and induction of tumor cell apoptosis | IL-2, IL-6, TNF-α, Bax, Bcl-2 | 400 mg/kg | [54] |
|  | Cell: SMMC-7721, Huh-7 cell  Animal: BALB/C mice | Relief of immune suppression and inhibition of tumor cell proliferation | miR-133a-3p, MSN, PD-L1 | 400 mg/kg | [55] |
|  | Cell: H22 cell | Inhibition of hepatocellular carcinoma cell viability and migration, and induction of apoptosis | Notch1, Bcl-2, BAX, caspase-3, caspase-8, E-cadherin, MMP-9, COX-2 | 1 mg/ml | [56] |
|  | Cell: Hep3B, L02 cell  Animal: Male BALB/c nude mice | Enhancement of doxorubicin-induced endoplasmic reticulum stress and tumor cell apoptosis | p-PERK, OGT, OGA, RL2, CTD110.6, Caspase-3, Bim, Bax, CHOP | 10 mg/l, 2 mg/kg | [57] |
|  | Cell: HepG2 cell | Induction of intracellular ROS accumulation leading to apoptosis | Cytochrome c, Bax, Bcl-2 | 400 μg/ml | [58] |
|  | Cell: HepG2 cell | Inhibits tumor cell proliferation, induces S-phase cell cycle arrest, and triggers apoptosis via the mitochondrial pathway | Cytochrome c, Bax, Bcl-2 | 800 μg/ml | [59] |
| Colorectal cancer | Animal: male C57BL/6 J mice | Suppresses cancer cell proliferation and enhances immune function | STAT3, Gal-3 | 100 mg/kg, 200 mg/kg, and 400 mg/kg | [75] |
|  | Cell: HCT-116 | Inhibits tumor cell proliferation | NA | NA | [76] |
|  | Cell: MC38  Animal: C57BL/6 mice | Promotes the activation and maturation of dendritic cells | CD8^+^, CD4^+^ T cells | NA | [77] |
| Gastric cancer | Cell: MGC-803 | Inhibits tumor cell proliferation and induces tumor cell apoptosis | Bax, Bcl-2, cytoplasmic Cytochrome c, caspase-9 | 800 µg/mL | [93] |
|  | Cell: AGS | Enhances the anti-gastric cancer effect of apatinib by modulating autophagy and apoptosis | VEGFR-2, MMP-9, LC-3, p-AKT, | 200 μg/ml | [94] |
|  | Cell: SGC-7901, SGC-7901/ADR cells | Enhances the pro-apoptotic effect of adriamycin on gastric cancer cells and significantly increases chemosensitivity | p-AMPK, caspase-3 | 50, 100, 200 µg/mL | [95] |
| Pancreatic cancer | Cell: ASPC-1, PANC-1 | Enhances the antitumor activity of apatinib against pancreatic cancer cells | VEGFR-2, MMP-9, Bax, Bcl-2, p-ERK, p-AKT, LC3 | 200, 400, 600 µg/mL | [100] |
